# Novel *PLCZ1* compound heterozygous mutations indicate gene dosage effect involved in total fertilisation failure after ICSI

**DOI:** 10.1530/REP-23-0466

**Published:** 2024-09-16

**Authors:** Qing Li, Juncen Guo, Gelin Huang, Nan Wu, Su Chen, Jing Dai, Xueguang Zhang, Guohui Zhang, Weiwei Zhi, Jierui Yan, Rui Zheng, Fei Yan, Zheng Yan, Ling Wu, Sixian Wu, Zhiliang Ji, Jiuzhi Zeng, Ge Lin, Bin Li, Wenming Xu

**Affiliations:** 1 Department of Obstetrics/Gynecology, Joint Laboratory of Reproductive Medicine (SCU-CUHK), Key Laboratory of Obstetric, Gynecologic and Pediatric Diseases and Birth Defects of Ministry of Education, West China Second University Hospital, Sichuan University, Chengdu, China; 2 State Key Laboratory of Cellular Stress Biology, School of Life Sciences, National Institute for Data Science in Health and Medicine, Xiamen University, Xiamen, Fujian, PR China; 3 Institute of Reproductive and Stem Cell Engineering, School of Basic Medical Science, Central South University, Changsha, China; 4 Key Laboratory of Reproductive Medicine, Sichuan Provincial Maternity and Child Health Care Hospital, Chengdu, China; 5 Department of Assisted Reproduction, Shanghai Ninth People's Hospital, Shanghai Jiaotong University School of Medicine, Shanghai, China

## Abstract

**In brief:**

*PLCZ1* mutations are related to total fertilisation failure (TFF) after intracytoplasmic sperm injection (ICSI), characterised by abnormal oocyte oscillations. The novel *PLCZ1* compound heterozygous mutations reported by this study were associated with TFF after ICSI, with one of the mutations indicating a gene dosage effect.

**Abstract:**

Oocyte activation failure is thought to be one of the main factors for total fertilisation failure (TFF) after intracytoplasmic sperm injection (ICSI), which could be induced by abnormal calcium oscillations. Phospholipase C zeta (PLCZ), a sperm factor, is associated with Ca^2+^ oscillations in mammalian oocytes. To date, some mutations in *PLCZ1* (the gene that encodes PLCZ) have been linked to TFF, as demonstrated by the observed reduction in protein levels or activity to induce Ca^2+^ oscillations. In this study, normozoospermic males whose sperms exhibited TFF after ICSI and their families were recruited. First, mutations in the *PLCZ1* sequence were identified by whole exome sequencing and validated using Sanger sequencing. Then, the locations of *PLCZ1*/PLCZ and the transcript and protein levels in the sperm of the patients were studied. Subsequently, *in vitro* function analysis and *in silico* analysis were performed to investigate the function–structure correlation of mutations identified in *PLCZ1* using western blotting, immunofluorescence, RT-qPCR, and molecular simulation. Ca^2+^ oscillations were detected after cRNA microinjection into MII mouse oocytes to investigate calcium oscillations induced by abnormal PLCZ. Five variants with compound heterozygosity were identified, consisting of five new mutations and three previously reported mutations distributed across the main domains of PLCZ, except the EF hands domain. The transcript and protein levels decreased to varying degrees among all detected mutations in *PLCZ1* when transfected in HEK293T cells. Among these, mutations in M138V and R391* of PLCZ were unable to trigger typical Ca^2+^ oscillations. In case 5, aberrant localisation of PLCZ in the sperm head and an increased expression of PLCZ in the sperm were observed. In conclusion, this study enhances the potential for genetic diagnosis of TFF in clinics and elucidates the possible relationship between the function and structure of PLCZ in novel mutations.

## Introduction

Early embryonic development is a complex process, including oocyte activation (OA), pronucleus formation, the release of the cortical granules, the extrusion of the second polar body, and other biological events (Dozortsev 1995). OA, the first stage of embryonic development in mammals, is initiated by a series of characteristic calcium (Ca^2+^) oscillations after sperm–oocyte fusion. Failure in OA results in the absence of normally fertilised zygotes due to an inability to induce Ca^2+^ spikes in oocytes (Campos *et al.* 2023). Additionally, embryonic arrest (also called zygote arrest) may occur owing to unclear factors during the stages of embryo cleaving (Lin *et al.* 2023). *In vitro fertilisation* (IVF) and intracytoplasmic sperm injection (ICSI) are the most commonly used assisted reproductive technologies for infertile couples. For male-infertile couples, especially patients with oligospermia, ICSI is a revolutionary technology. However, total fertilisation failure (TFF) after IVF and ICSI is a persistent problem in fertility clinics. This refers to the failure to form normally fertilised zygotes (two-pronucleus (2 PN) zygotes) at 16–19 h post insemination, excluding cases when the female yields a low number of mature eggs (fewer than three oocytes) (Flaherty 1998). TFF may occur in up to 5–10% of IVF cycles and 3% of ICSI cycles (Mahutte & Arici 2003). The most common aetiology of TFF is oocyte activation failure (OAF). Abnormal fertilisation and zygote arrest are also common problems in TFF (Torra-Massana *et al.* 2023).

Phospholipase C zeta (PLCZ), a testis-specific protein initially identified from sperm extracts (Saunders 2002), could trigger Ca^2+^ oscillations in oocytes that are similar to those induced by gamete fusion (Saunders 2002, Fujimoto *et al.* 2004, Kurokawa *et al.* 2005, Nomikos *et al.* 2013). Injection of *PLCZ1* (the gene that encodes PLCZ) cRNA and PLCZ recombinant protein into human or mouse oocytes could induce Ca^2+^ oscillations and initiate blastocyst formation (Saunders 2002, Kouchi *et al.* 2004, Rogers *et al.* 2004, Kouchi *et al.* 2005, Yoon *et al.* 2012). *PLCZ1* variants are associated with the failure to trigger normal calcium oscillations (Heytens *et al.* 2009, Torra-Massana *et al.* 2019, Torra-Massana *et al.* 2023). Because PLCZ plays a vital role in OA, *PLCZ1* is considered a key gene associated with TFF (Dai *et al.* 2020). Sperm from the *Plcz1* KO mouse generated using the CRISPR/Cas9 system failed to trigger Ca^2+^ spikes in ICSI and induced atypical Ca^2+^ spikes following IVF (Hachem *et al.* 2017, Nozawa *et al.* 2018). However, polyspermic fertilisation was found in fertilised oocytes collected after IVF using sperm from *Plcz1* KO mice and the oviducts after crossing with these transgenic males (Hachem *et al.* 2017, Nozawa *et al.* 2018). Recent studies have reported that *PLCZ1* mutations are also associated with early embryonic arrest and polyspermy (Lin *et al.* 2023, Peng *et al.* 2023), indicating that complex relationships between *PLCZ1* mutations and functional alterations warrant detailed investigation.

PLCZ is a member of the phosphoinositide-specific phospholipase C family, comprising two functional domains (the X catalytic domain and the Y catalytic domain), two regulatory domains (N-terminal of the EF-hands domain and C-terminal of the C2 domain), and a region called the X–Y linker region, as described by UniProt. The EF-hands domain is related to the calcium-binding ability of PLCZ. PLCZ hydrolyses phosphatidylinositol 4,5-bisphosphate (PIP2) into inositol triphosphate (IP3) and diacylglycerol (DAG) and finally evokes intracellular calcium oscillations within the oocyte via the IP3 Ca^2+^ signalling pathway (Swann *et al.* 2006). According to previous studies, mutations in the X catalytic domain, Y catalytic domain, X–Y linker region, and C2 domain of *PLCZ1* were proved to be pathogenic or potentially pathogenic, while mutations in the EF-hands domain are likely to be benign or their impact remains unclear (Supplemental Table S1, see section on supplementary materials given at the end of thisarticle). These mutations may be detrimental to the enzyme’s catalytic function (Mu *et al.* 2020), affecting the transcription of *PLCZ1* or the generation of Ca^2+^ transients in oocytes (Yan *et al.* 2020, Zhao S 2023). Additionally, mutations in the C2 domain, located at the interface with the catalytic domain, such as L576P, could also affect the enzyme’s catalytic function (Yuan *et al.* 2020). As for the EF-hands domain and its nearby regions, functional analysis in these regions was limited in previous studies (Supplemental Table S1). p.I120M, a mutation located between the EF-hands domain and the X catalytic domain according to Uniprot or the EF-hands domain described by an article (Torra-Massana *et al.* 2019), was reported to have no effect on OA based on *in silico* and functional analysis. However, patients with this mutation in heterozygous form experienced TFF, suggesting the need for further exploration of mechanisms linking structure and function (Torra-Massana *et al.* 2019). Furthermore, in normal sperm, PLCZ is found in the perinuclear theca located in the sperm head, specifically in the acrosomal/equatorial region (AcEq), equatorial region alone (Eq), and equatorial/post-acrosomal region (EqPa) (Grasa *et al.* 2008). Previous studies have indicated that sperms that cannot trigger OA exhibit abnormal localisation of PLCZ (Grasa *et al.* 2008, Torra-Massana *et al.* 2019). Therefore, the relationship between protein expression, localisation, and function of *PLCZ1*/PLCZ needs further clarification.

Here, five patients with *PLCZ1* mutations experiencing TFF were recruited. Eight mutations in *PLCZ1* were identified, including five novel mutations. Their functional and conformational alterations were investigated using *in silico* and *in vitro* function analysis. A newly reported mutation was localised in a region between the EF-hands domain and X catalytic domain, and its function and structure alteration were reported. Furthermore, a novel variant in compound heterozygosity with an elevated protein expression level was reported for the first time. This study elucidated the pathogenic mechanism of *PLCZ1* variation and the genetic diagnosis of male infertility due to TFF, especially in the normozoospermic sperm sample.

## Materials and methods

### Clinical samples

Patients with TFF after ICSI due to male-related factors and their family members were recruited from West China Second University Hospital, Sichuan Provincial Maternity and Child Health Care Hospital, and Shanghai Ninth People’s Hospital. Healthy Chinese volunteers without any evidence of infertility were recruited from the Physical Examination Centre at West China Second University Hospital. All patients and their family members provided written informed consent before the study. This study followed the tenets of the Declaration of Helsinki and was approved by the Ethical Review Board of West China Second University Hospital, Sichuan University (approval no. 2020-031).

### Whole exome sequencing and Sanger sequencing

Peripheral blood samples were obtained from the patients and their family members. The genomic DNA was isolated in accordance with the instructions provided by DNeasy Blood & Tissue Kits (69504, Qiagen). Next-generation sequencing was then conducted using the SureSelectXT Human All Exon Kit (5190-8864, Agilent) and Illumina HiSeq X-TEN. The reads were aligned to the human genome reference assembly (UCSC GRCh37/hg19) with BWA 0.7.9a from the BWA-MEM algorithm. These candidate variants in *PLCZ1*, identified using whole exome sequencing (WES), were validated by Sanger sequencing. The PCR primers are listed in Supplemental Table S2.

### Bioinformatic analysis and molecular modelling

The effect of variants was predicted by Mutation Taster (https://www.genecascade.org/MutationTaster2021), PolyPhen-2 (http://genetics.bwh.harvard.edu/pph2/), and SIFT (https://sift.bii.astar.edu.sg/) softwares. Moreover, the allele frequencies were determined using the ExAC, 1000 Genomes, gnomAD, and ALFA databases.

The three-dimensional structure of PLCZ was predicted by the AlphaFold application (Jumper *et al.* 2021). The 3D coordinates of PLCZ are available in the Protein Data Bank (PDB) format from the following link: https://www.rcsb.org/structure/AF_AFQ86YW0F1 in the PDB database (Berman *et al.* 2000) or from the AlphaFold Protein Structure Database (ebi.ac.uk). For the structural simulation of amino acid mutations at the protein unit point, we used the PyMOL software (WL 2002) to identify the mutated proteins (PLCZR391* and PLCZM138V) and the GROMACS software (Abraham *et al.* 2015) to simulate molecular dynamics for 50 ns. Graphs and figures were generated using the QtGrace software (v0.2.6) to visualise the differences between *PLCZ1* and its mutations.

### Papanicolaou staining

The sperm cells were fixed in 4% paraformaldehyde after washing in phosphate-buffered saline (PBS) and spotted onto slides. The dried sperm smears were immersed in 80% ethanol, 50% ethanol, and water for 30 s, respectively. Haematoxylin staining was performed for 10 min, and the smears were then rinsed with water for 30 s. The smears were differentiated using a hydrochloric acid–alcohol solution for several seconds and then rinsed in running water for 5 min until they became blue again. Subsequently, the smears were immersed in 50%, 75%, and 95% ethanol for 30 s, 30 s, and 15 min, respectively. Orange G staining was performed for 1 min. The smears were then immersed in 95% ethanol thrice for 30 s each time. Afterwards, the smears were stained with an EA-50 staining solution for 1 min. The smears were immersed in 95% ethanol twice for 30 s and 100% ethanol twice for 15 s each time. Finally, an oil seal was performed with cedarwood oil. Images were obtained with an inverted research microscope (ECLIPSE Ti2-U, Nikon).

### Transmission electron microscopy

Sperms collected from patients and healthy volunteers were washed with SpermRinse™ (10,101, Vitrolife, Kungsbacka, Sweden), fixed in 3% glutaraldehyde and postfixed with 1% OsO_4_. The specimens were embedded in Epon 812 and sliced. The ultrathin sections were stained with uranyl acetate and lead citrate. Images were obtained using a transmission electron microscopy (TEM) camera (TECNAI G2 F20, Philips), operating at a voltage of 120 kV.

### Plasmid construction and cell culture

The plasmids of PLCZ^WT^ were manufactured by Vigene Biosciences (Jinan, China). A fast mutagenesis system (FM111, Transgen, Beijing, China) was used to generate mutant plasmids following the manufacturer’s protocol by using PLCZ^WT^ constructs as templates. The primers used are listed in Supplemental Table S3. Transformed human embryonic kidney cells (HEK293T) were cultured in DMEM (11965092, Gibco) with 10% fetal bovine serum (FBS) (F8318, Sigma-Aldrich) under standard conditions. HEK293T cells were transfected with plasmids of PLCZ^WT^ and mutant plasmids using the jetPEI Transfection Kit (Poly-Plus Transfection, Strasbourg, France).

### Immunofluorescence staining

The sperm from patients and healthy volunteers was fixed with 4% paraformaldehyde after washing in PBS and spotted onto slides. After permeabilizing the sperm with 0.3% Triton X-100 in PBS for 30 min at room temperature, they were blocked in 10% bovine serum albumin (A1933, Sigma-Aldrich) for 1 h and incubated overnight at 4°C with PLCZ antibody (1/1000, ab181816, Abcam). 1× PBS buffer was used to wash the smears, followed by incubation with Alexa Fluor 488 (1:1000, A21206, Thermo Fisher Scientific) and DAPI (D9542, Sigma-Aldrich) dyes for 1 h. Fluorescence imaging was performed using a confocal microscope (FV3000, Olympus).

### Western blotting analysis

To extract total proteins, the sperm cells were washed in PBS and then resuspended in Radio Immunoprecipitation Assay (RIPA) lysis buffer (P0013C, Beyotime, China) supplemented with Halt™ Protease Inhibitor Cocktail (78,425, Thermo Fisher Scientific). These total protein extractions were mixed with SDS sample loading buffer (P0015, Beyotime, Haimen, China) and boiled for 10 min at 95˚C. The protein extracts were loaded into a 10% SDS–PAGE (PG112, Epizyme Scientific, Shanghai, China). The separated proteins were then transferred onto 0.45 m PVDF membranes (Millipore). The membranes loaded with proteins were blocked for 1 h with TBST containing 5% milk and incubated with anti-Flag antibody (1/1000, PA5-98556, Invitrogen) and subsequently with horseradish peroxidase (HRP)-conjugated secondary antibodies for 1 h. Immunoreactivity was detected using an ECL chemical substrate (WBKLS0100, Millipore).

### cRNA preparation

PCR was employed to amplify the DNA of PLCZ^WT^ and mutants, using the plasmids of PLCZ^WT^ and mutants as templates purified with the Universal DNA Purification Kit (DP214-03, TIANGEN, Beijing, China). The primers used for amplification were the following: 5′-TAATACGACTCACTATAGGGATGGAAATGAGATGGTTTTTGTCAAAGATTCAGGATG-3′ (forward) and 5′-TTATCTGACGTACCAAACATAAACAAACAGTG-3′ (reverse). cRNA was then synthesised by the T7 High Yield RNA Transcription Kit (E131, Novoprotein, Suzhou, China) and purified by RNA Clean & Concentrator-5 (R1015, Zymo Research, Irvine, CA, USA).

### Ca2+ monitoring

MII mouse eggs were obtained as described below. Briefly, 3-week-old female mice were superovulated by injection of PMSG (Hangzhou Animal Medicine Factory, Hangzhou, China) and HCG (Hangzhou Animal Medicine Factory, China). The eggs were collected 14–16 h after injection of HCG and maintained in M2 medium (EmbryoMax M2 Medium, MR-015, Sigma) supplemented with mineral oil at 37°C and 5% CO_2_; the medium was prebalanced overnight in an incubator. The eggs were incubated with Fluo-3 AM (S1056, Beyotime) according to the protocol, and an equal volume – about 3–5% of the egg volume – of PLCZ^WT^ and mutant cRNA at a concentration of 600 ng/μL were injected. Ca^2+^ levels were monitored for 3 h at an excitation wavelength of 488 nm employing a laser scanning confocal microscope (LSM900 + Airyscan2, Zeiss).

### Statistical analysis

All data were analysed by GraphPad Prism 9.0 software and expressed as the means ± s.e.m . The two-sided Student’s *t*-test was used to compare two independent groups. One-way ANOVA was used to analyse the data in more than two groups. *P* < 0.05 was considered statistically significant.

## Results

### Clinical data

Five couples who had experienced male infertility for more than 2 years were recruited. As shown in Table 1, the sperm-related parameters of the patients, especially the morphology of normal sperm and the total sperm count, significantly exceeded the lower reference limits set by the WHO guidelines as shown in Table 1. However, despite these findings being compatible with spontaneous conception, TFF occurred following ICSI. Except for family 2, normally fertilised zygotes were absent in the ICSI cycles of the five couples. In family 2, despite the presence of four fertilised oocytes after ICSI, no post-cleavage embryos were observed. Remarkably, when rescued with AOA-ICSI, post-cleavage embryos were observed in all the AOA-ICSI cycles, resulting in the birth of a girl and a boy in family 3 and family 4, respectively. As for family 5, nine MII oocytes were retrieved from the female. Two oocytes developed viable eight-cell embryos with AOA-ICSI but failed to result in pregnancy. The outcomes of ICSI and AOA-ICSI are shown in Table 2. Overall, the clinical outcomes of assisted reproduction technologies, such as ICSI and AOA-ICSI, indicate that sperm-induced OAF might have occurred among the five families.

**Table 1:** Semen parameters of patients mutated in *PLCZ1*. Lower and upper reference limits are shown according to the World Health Organisation standards (WHO 2010) (Cooper *et al.* 2010).

Semen parameters	Patient 1	Patient 2	Patient 3	Patient 4	Patient 5	Reference values
Semen volume (mL)	2.7	1.7	2.5	3	4.3	≥1.5
Sperm concentration (10^6^/mL)	20.1	90.1	40.5	235	96	≥15.0
Progressive motility (%)	20	76.3	46	82.7	44	≥32.0
Morphologically normal sperm (%)	3	6	6	8	5	≥4
Sperm with head defect (%)	–	–	–	–	95	–
Sperm with tail defect (%)	–	–	–	–	7	–

**Table 2:** Clinical characteristics of five patients undergoing IVF, ICSI, or AOA-ICSI cycles.

Case	Age (years)		PIH (years)	Cycle	Number of							IR	Live birth
	Male	Female			Oocytes injected	Fertilized oocytes	Cleavage embryos	Six cells	Eight cells	TR cycles	TE/cycle		
Patient 1	28	29	3	ICSI	17	0	–	–	–	–	–	–	–
Patient 2	34	30	6	ICSI	44	4	–	–	–	1	2	0	0
Patient 3	33	35	10	ICSI	12	0	–	–	–	–	–	–	–
				AOA-ICSI	12	8	11	2	9	1	2	1	1F
Patient 4	34	33	10	ICSI	2	0	–	–	–	–	–	–	–
				AOA-ICSI	2	2	1	0	1	–	–	–	–
				AOA-ICSI	7	6	6	6	6	1	2	1	1M
Patient 5	27	–	4	IVF	11	4	2	–	–	2	2	–	–
				ICSI	9	0	–	–	–	–	–	–	–
				AOA-ICSI	9	2	2	–	2	1	1	0	–

IR, implantation rate; PIH, primary infertility history; TE, transferred embryos; TR, transfer.

### Mutation profiles in *PLCZ1* identified in the TFF patients

**Figure 1:**
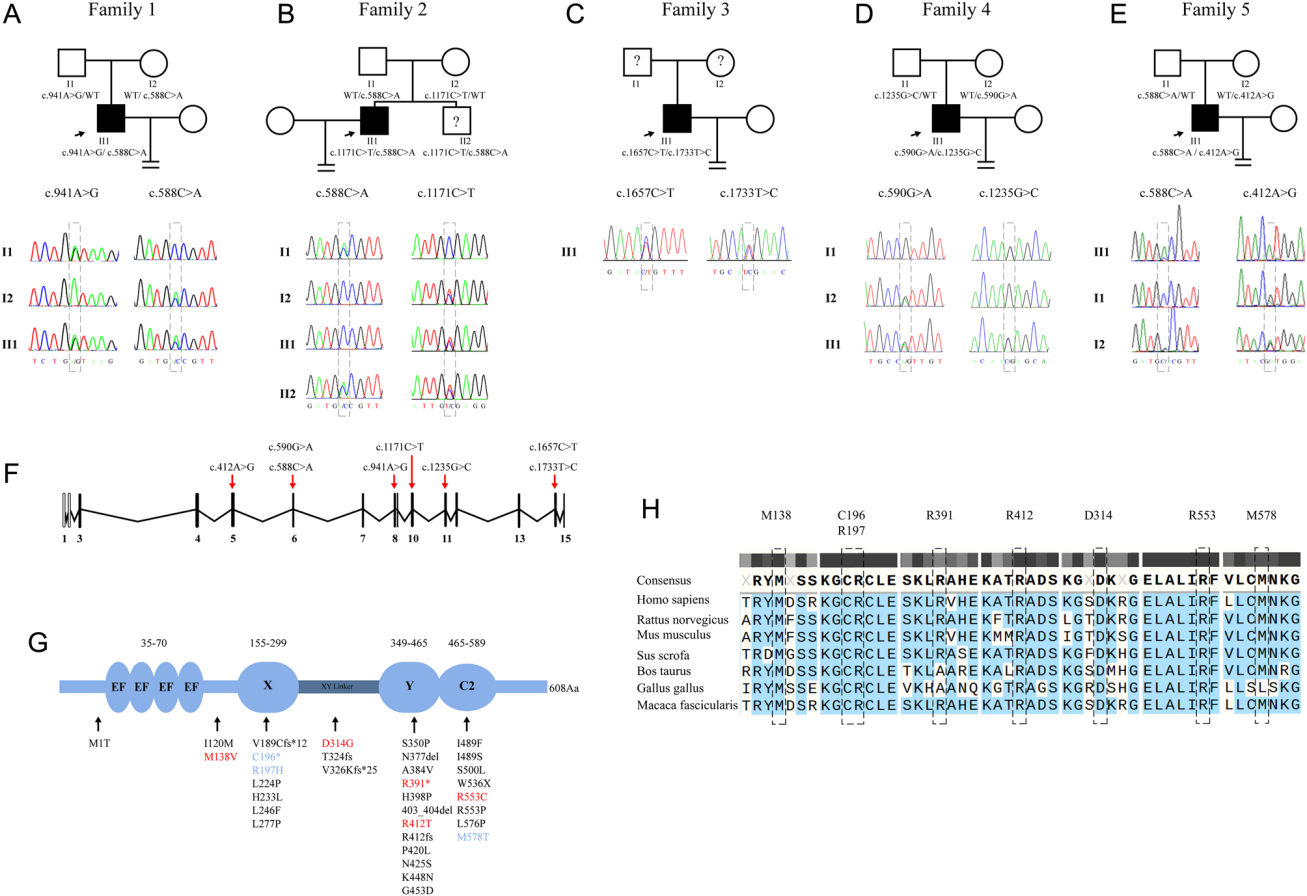
Variants in *PLCZ1* identified in five patients suffering from TFF. (A–E) Pedigrees of the five families with *PLCZ1* mutations. Males were denoted by squares, and females were denoted by circles. The probands were indicated by black arrows. (F) The positions of the mutations in the structure of the *PLCZ1* gene (NCBI accession number is NM_033123.3). (G) Schematic illustration of the domains in PLCZ and the positions of the mutations in the protein (UniProt ID: Q86YW0). Five novel mutations identified in our study were highlighted in red. Three mutations highlighted in blue were reported by previous studies but identified in this study. The others in black were mutations found in TFF patients reported yet. (H) The conservation of the mutated amino acids in seven different species.

Sperm-induced OAF was suspected as the primary factor causing infertility among the five families. Previous studies have highlighted that mutations in *PLCZ1* play a vital role in the OAF of TFF. Therefore, WES combined with Sanger sequencing was performed to identify mutations in the *PLCZ1* gene of males and their family members. As shown in Fig. 1 and Table 3, eight different mutations were identified, and all of them were determined to be compound heterozygous mutations in *PLCZ1* among the enrolled patients (Fig. 1F), among which five mutations were newly reported: c.941A>G (p.D314G), c.1171C>T (p.R391*), c.1657C>T (p.R553C), c.1235G>C (p.R412T), and c.412A>G (p.M138V). Sequencing of their parents’ DNA showed that most of them were the carriers of these compound heterozygous mutations except family 3. All mutations exhibited extremely low allele frequencies in the 1000 genomes, ALFA, ExAC, and genomAD databases. Among these identified mutations, p.M138V, a missense mutation located between the EF-hands domain and the X catalytic domain, was predicted to be deleterious by SIFT. p.C196* and p.R197H were stop-gain and missense mutations in the X catalytic domain, respectively. p.D314G was identified in the X–Y linker region and predicted to be deleterious by SIFT. p.R391* and p.R412T were located in the functional domain of PLCZ: the Y catalytic domain, while p.R553C and p.M578T were in the regulatory region: the C2 domain. These mutations were found to be highly conserved across species in mammals according to Clustal Omega analysis (Fig. 1H).

**Table 3:** Mutations in *PLCZ1* identified in the TFF patients. All data are based on GRCh37/hg19, NM_033123.3 (NCBI accession no.), and Q86YW0 (UniProt ID).

Case/MT site	Variation (dbSNP)	Exon	Domain	Variant allele	MT type	Allele frequency in human population				Function prediction		
						1000 G	gnomAD	ALFA	ExAC	PP2	SIFT	MTT
Patient 1												
c.941A>G (p.D314G)	12:18854634	8	X-Y L	Het	MS	NA	NA	NA	NA	Benign	DEL	Benign
c.588C>A (p.C196*)	12:18865902	6	X	Het	SG	0.000156/1	0.000016/4	NA	0.000016/2	NA	DEL	DEL
Patient 2												
c.1171C>T (p.R391*)	12:18852731	10	Y	Het	NS	NA	0.00001994/5	0.00006/4	0.0000166/2	NA	DEL	DEL
c.588C>A (p.C196*)	12:18865902	6	X	Het	SG	0.000156/1	0.000016/4	NA	0.000016/2	NA	DEL	DEL
Patient 3												
c.1657C>T (p.R553C)	12:18837148	14	C2	Het	MS	NA	0.000057/8	NA	NA	PRD	DEL	Benign
c.1733T>C (p.M578T)	12:18837072	14	C2	Het	MS	NA	NA	NA	0.000017/2	POD	DEL	DEL
Patient 4												
c.590G>A (p.R197H)	12:18865900	6	X	Het	MS	NA	0.000007/1	0./0	0.000016/2	PRD	DEL	DEL
c.1235G>C (p.R412T)	12:18849140	11	Y	Het	MS	NA	NA	NA	NA	PRD	DEL	DEL
Patient 5												
c.588C>A (p.C196*)	12:18865902	6	X	Het	SG	0.000156/1	0.000016/4	NA	0.000016/2	NA	DEL	DEL
c.412A>G (p.M138V)	12:18872522	5	–	Het	MS	NA	NA	NA	0.000027/3	Benign	DEL	Benign

Del, deleterious; MS, missense; MT, mutation; MTT, mutation taster; NS, nonsense; POD, possibly damaging; PP2, PolyPhen-2; X-Y L, X-Y Linker; PRD, probably damaging; SG, stop gain; 1000 G, 1000 Genomes.

### Deleterious effects of identified mutations in *PLCZ1* on transcriptional level and protein expression

**Figure 2:**
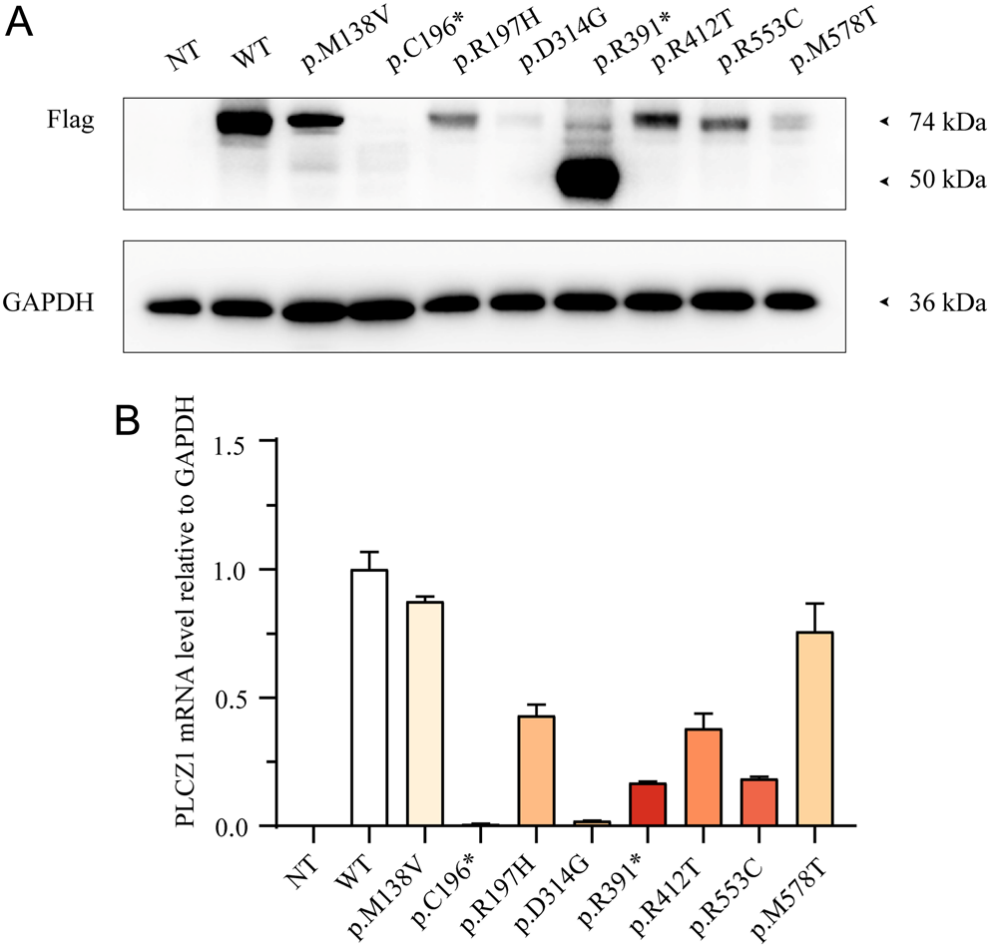
*In vitro* function analysis of the identified mutations by transfecting HEK293T cells with constructs of *PLCZ1*. (A) The effects of the identified mutations in the translation of *PLCZ1* by western blotting. (B) The effects of the identified mutations in the transcription of *PLCZ1* by RT-qPCR. NT. The sample that transfected HEK293T cells with the vector of plasmid.

Eukaryotic expression vectors of wild-type (Flag-WT-*PLCZ1*) and mutant *PLCZ1* (Flag-mut-*PLCZ1*), including the identified mutations, were constructed and transfected into HEK293T cells. *In vitro* function analysis was conducted using western blotting and RT-qPCR to reveal the possible impact on protein expression and transcription levels, as shown in Fig. 2. Notably, decreased levels of proteins (without strip trailing) and mRNA were observed in the group of p.C196*, indicating a deleterious effect on transcription and protein expression. The proteins comprising the p.R197H, p.D314G, p.R412T, p.R553C, and p.M578T groups exhibited a significant reduction in both molecular weight and transcription levels. In the group of p.R391*, a truncated protein was observed. Moreover, increased protein levels and a slight reduction in mRNA levels were demonstrated. Regarding p.M138V, a slight reduction in protein level was observed according to WB, and RT-qPCR did not identify any statistically significant difference between the p.M138V and WT groups. Considering that the patient in family 5 exhibited compound heterozygosity for a variant (c.588C>A: p.C196*; c.412A>G: p.M138V), a functional analysis of the mutant p.M138V relating to its ability to trigger normal Ca^2+^ oscillations would be carried out in a follow-up experiment.

### Novel R391* and M138V mutations alter PLCZ conformation

**Figure 3:**
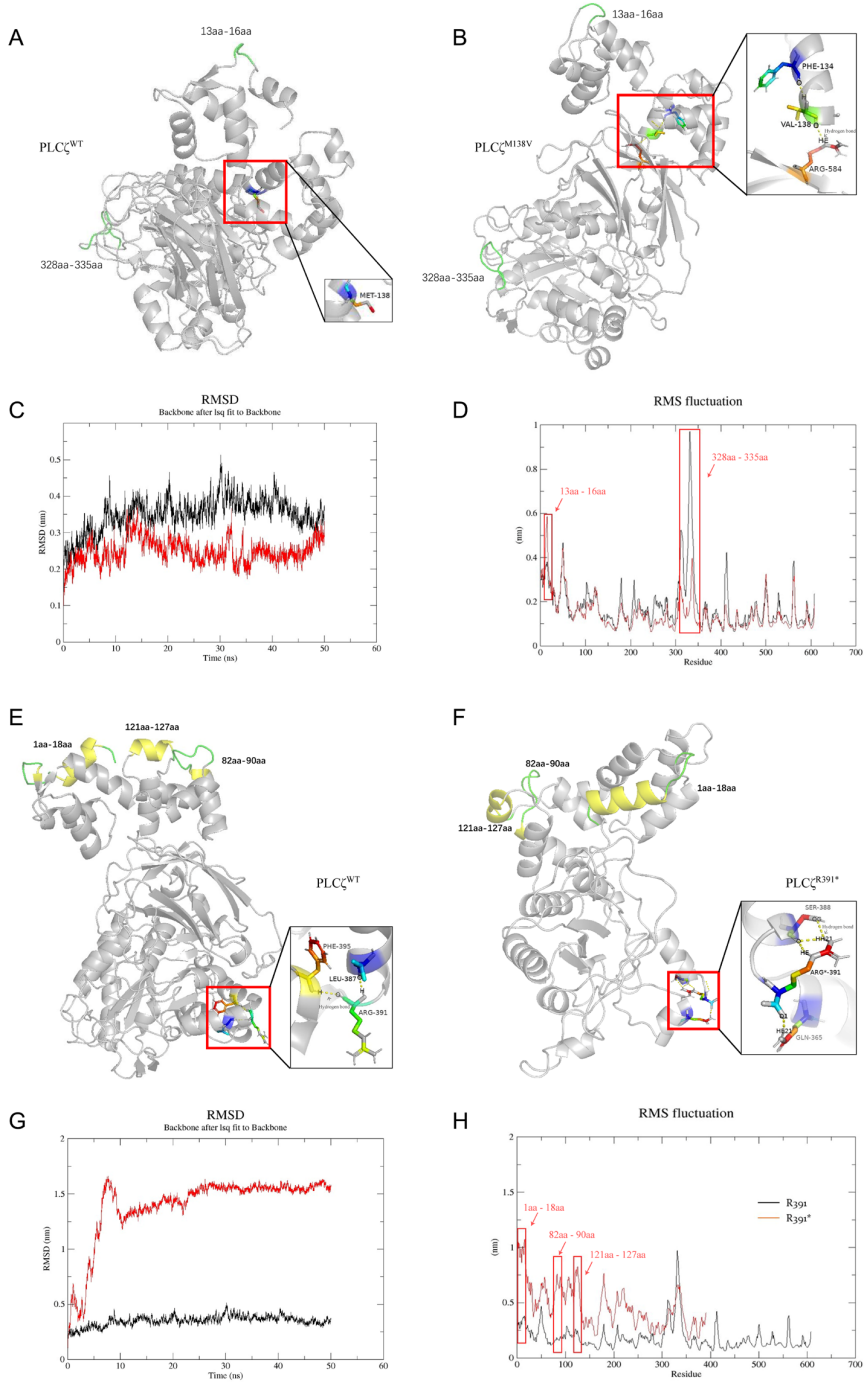
Molecular dynamic simulation of the effect of the mutations (p.R391* and p.M138V) on the conformation of PLCZ. (A and B) The predicted 3D model of PLCZ^WT^ and PLCZ^M138V^. Hydrogen bondings formed at 50 ns of simulation were shown in the enlarged red boxes. A new hydrogen bonding was exhibited in PLCZ^M138V^ between residues valine 138 and arginine 584. (C) The alterations to root mean square deviation (RMSD) in PLCZ^WT^ and PLCZ^M138V^ depending on the time for molecular dynamic simulation. (D) Root mean square deviation fluctuation (RMSF) of PLCZ^WT^ and PLCZ^M138V^. The value of res-RMSF between the two regions – 13aa–16aa and 328aa–335aa – was quite different. (E and F) The predicted 3D model of PLCZ^WT^ and PLCZ^R391*^. Hydrogen bonds formed between amino acids at 50 ns of simulation were shown in enlarged red boxes. The residues for hydrogen bond formation (with arginine 391) and the total amount of hydrogen bonds were diverged in PLCZ^WT^ and PLCZ^R391^*. (G) The alterations to RMSD in PLCZ^WT^ and PLCZ^R391*^ depending on the time for molecular dynamic simulation. (H) RMSF of PLCZ^WT^ and PLCZ^R391*^.

To further investigate the impact of structural alteration on the expression change, a molecular dynamic simulation was performed on the mutations (p.R391* and p.M138V). Compared with PLCZ^WT^, PLCZ^M138V^ possessed a new hydrogen bond between residues V138 and R584 (Fig. 3A and B and Supplementary Figures 1 A and C). The value of Cα-RMSD for *PLCZ1* ^WT^ was comparable to that of PLCZ^M138V^ across various simulation times (Fig. 3C), which indicated that only a few alterations may have occurred in PLCZ^M138V^ compared to *PLCZ1* ^WT^. Moreover, the res-RMSF value showed obvious differences at two regions between PLCZ^WT^ and PLCZ^M138V^: 13aa–16aa and 328aa–335aa (Fig. 3D). As for p.R391*, significant differences were observed compared to PLCZ^WT^, including the amount of hydrogen bond and its bonding residues and the value of RMSD, which showed a huge structural alteration between PLCZ^WT^ and PLCZ^R391*^ (Fig. 3E and F and Figures SI B and D). Compared with PLCZ^WT^, PLCZ^R391*^ possessed fewer stable hydrogen bonding such as the hydrogen bonding between residues R391 and F395 and the hydrogen bonding between residues R391 and L387. A significant difference was shown in the res-RMSF value between PLCZ^WT^ and PLCZ^R391*^ at three regions: 1aa–8aa, 82aa–90aa, and 121–127aa (Fig. 3H). Together, the molecular dynamic simulation results showed a significant effect of the mutation on the conformational change of the proteins, which could affect protein stability and other aspects of protein function.

### Influence of R391* and M138V mutations on PLCZ-induced normal calcium oscillations

**Figure 4:**
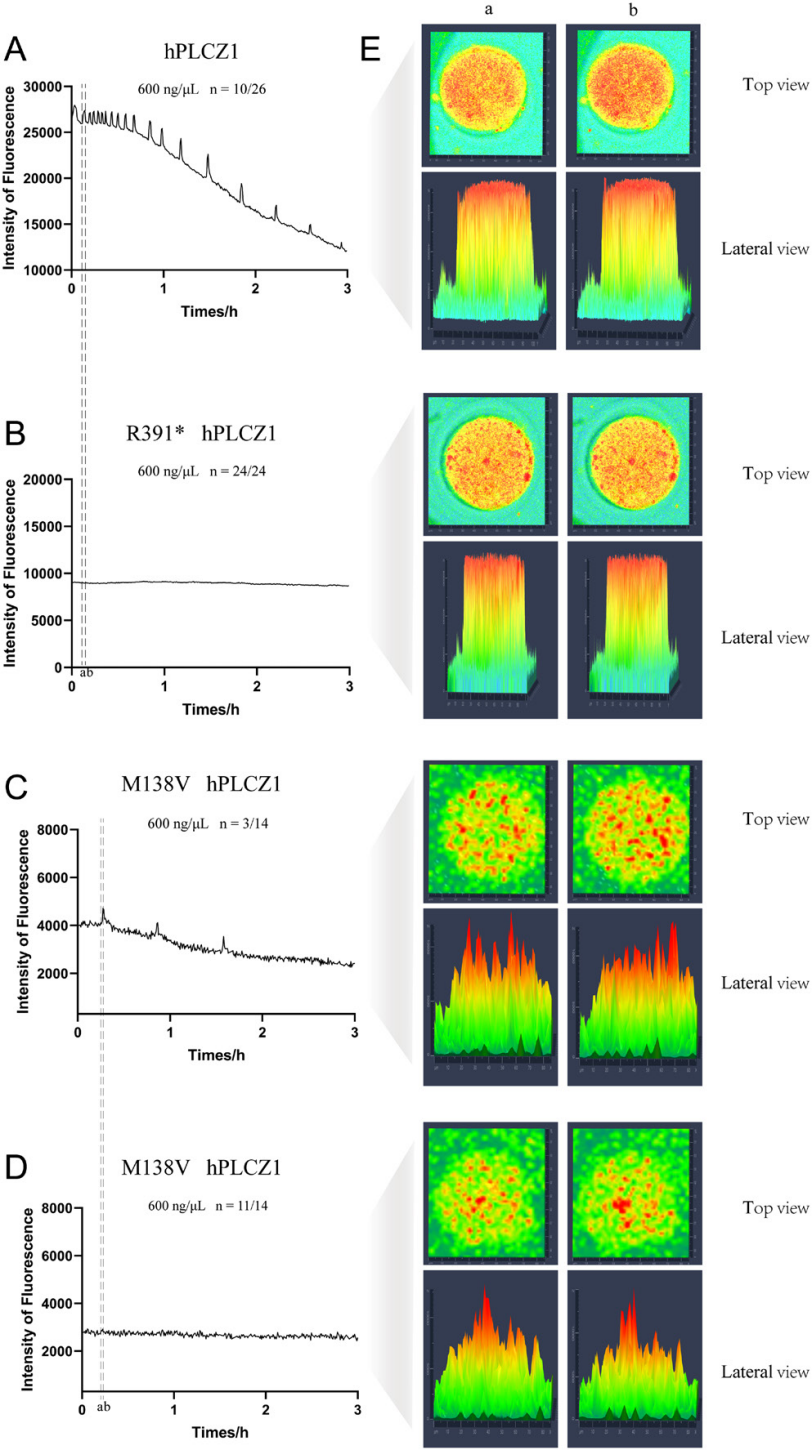
The R391* and M138V mutations influence the PLCZ function of inducing normal calcium oscillations. (A–D) Ca^2+^ monitoring was performed by microinjecting cRNA of hPLCZ1^wt^, hPLCZ1^R391*^, and hPLCZ1^M138V^ into mouse MII oocytes at a concentration of 600 ng/μL. (A) hPLCZ1^wt^ cRNA initiated high-frequency oscillations in 10 out of 26 injected oocytes, with a characteristic pattern of decreasing frequency and intensity over time. (B) No spikes of oscillations were obtained by hPLCZ1^R391*^ cRNA injection (*n* = 24). (C and D) Ca^2+^ oscillations were detected in 3 out of 14 oocytes microinjected with hPLCZ1^M138V^ cRNA (*n* = 3/14). (E) Representative images demonstrate the effect of the mutations on the PLCZ calcium imaging pattern. The M138V mutant shows a more sparse and punctate calcium release pattern with larger dot clusters compared to the wild-type PLCZ. In contrast, the R391* mutant exhibits a significantly lower calcium signal intensity than the normal PLCZ, as evident from the lack of obvious changes in fluorescence intensity in the top and lateral views.

Ca^2+^ levels were monitored after microinjecting cRNAs of h*PLCZ1*^M138V^, h*PLCZ1*^R391*^, and h*PLCZ1*^wt^ into mouse MII oocytes to evaluate their corresponding enzymatic activity in inducing calcium spikes. h*PLCZ1*^wt^ cRNA (600 ng/μL) induced high-frequency Ca^2+^ oscillations in 10 out of 26 injected oocytes (Fig. 4A). Ca^2+^ oscillations decreased in frequency and intensity over time. As for the h*PLCZ1*^M138V^ and h*PLCZ1*^R391*^ groups, Ca^2+^ oscillations were detected in 3 out of 14 oocytes microinjected with h*PLCZ1*^M138V^ cRNA (600 ng/μL) (Fig. 4C and D). No spikes of oscillation were obtained in oocytes injected with h*PLCZ1*^R391*^ cRNA at the same concentration (Fig. 4B). A stronger signal and fluorescence intensity were detected at peaks of calcium oscillation than at valleys, as shown in Fig. 4. However, no obvious changes were observed in the images of fluorescence from eggs microinjected with h*PLCZ1*^R391*^ cRNA.

### Expression changes in variants on sperms of patient in family 5 indicates a gene dosage effect

**Figure 5:**
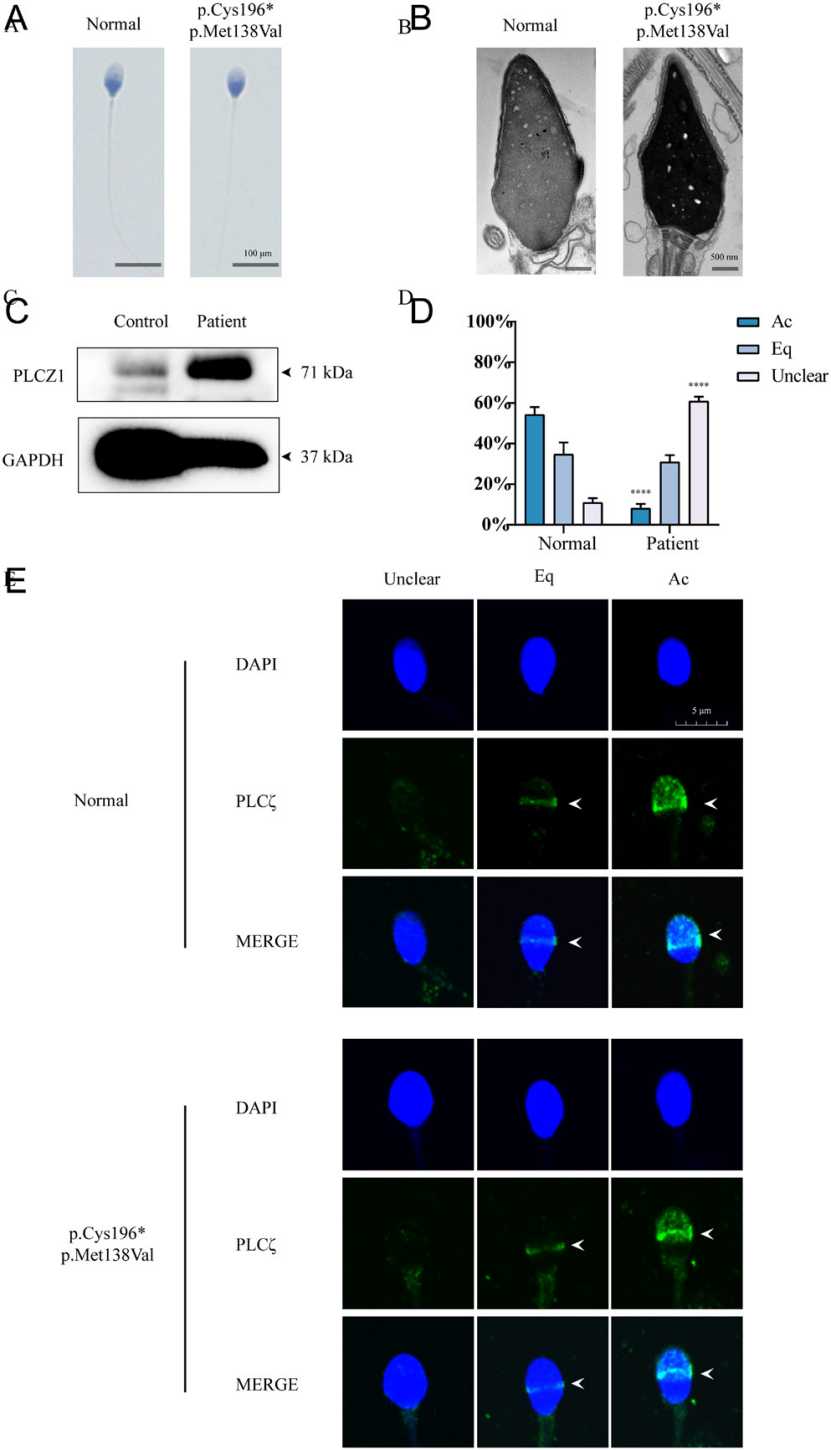
The deleterious effects of variants in the patient of family 5. (A) The morphology of sperms by Papanicolaou staining. (B) Ultrastructure of spermatozoa from the patient and control by TEM. (C) Comparison of protein levels of spermatozoa by western blotting. (D) The proportion of each pattern in normal and mutant sperm. (E) The localisation patterns of PLCZ. The white arrows showed the location of PLCZ. Eq, equatorial region; Ac, acrosomal region. Scale bar: (A) 100 μm; (B) 500 nm.

We investigated the morphology and ultrastructure of sperm from the patient in case 5 to determine the harmful impact of the variations in compound heterozygosity using Papanicolaou staining and TEM. No obvious abnormalities were observed in the sperm of the patient compared with the control (Fig. 5A and B). Interestingly, increased PLCZ levels were detected in the patient’s sperm using western blotting analysis (Fig. 5C), indicating a weak correlation between PLCZ content and TFF in case 5. The location of PLCZ in spermatozoa was evaluated by IF (Fig. 5D and E). Three categories of sperm regions were observed based on PLCZ locations: Eq (equatorial region), Ac (acrosomal region), and unclear region (regions that cannot be described clearly). Compared with the healthy volunteers, a significant difference was observed in the presence of certain variants (p. C196* and p. M138V) in the patient with respect to the proportion of sperm with PLCZ located at the Ac and unclear region. It is concluded that both the gene dosage and localisation were altered in the sperm sample of the fifth patient.

## Discussion

### Deleterious effects of identified mutations in *PLCZ1*

In this study, mutations were identified in almost all the regions of PLCZ sequences, excluding the EF-hands domain. A notably decreased level of protein expression and mRNA levels were observed in the group comprising p.C196*, p.R197H, p.D314G, p.R412T, p.R553C, and p.M578T, which revealed that mutations in these regions played a vital role in the transcription and protein expression of *PLCZ1*/PLCZ. However, the p.M138V mutation showed no significant impact on transcription or translation of *PLCZ1*/PLCZ in HEK293T cells but exhibited a decreased ability to trigger Ca^2+^ spikes when its cRNA was injected into mouse MII oocytes. The 3D structure of PLCZ^wt^ and PLCZ^M138V^ exhibited significant similarity during molecular dynamic simulation in this study. Some studies reported that the EF-hands domain regulates enzyme activity relating to regulating Ca^2+^ sensitivity and the ability to bind with membrane phospholipids. Alterations in the EF-hands domain required a higher Ca^2+^ concentration in the cytoplasm to induce Ca^2+^ oscillations and exhibited a decreased ability to bind to PI (4,5)P2. Future studies should focus on examining the relationship between the structure of PLCZ^M138V^ and its Ca^2+^ sensitivity and its ability to interact with membrane phospholipids. p.R391*, predicted to be deleterious by the SIFT and Mutation Taster software, resulted in reduced protein expression in HEK293T cells. A huge structural alteration between PLCZ^WT^ and PLCZ^R391*^ was revealed by *in silico* analysis. Furthermore, no PLCZ expression was detected in sperm with the homozygous nonsense variation p.C196* (Dai *et al.* 2020), which indicated that a functional impairment might occur due to the deletion of PLCZ in sperms with variation p.C196*. In case 5, the sperm of the male patient carrying a compound heterozygous variant (p.C196* and p.M138V) showed fertilisation failure after ICSI. During IVF, however, post-cleavage embryos formed, probably due to the presence of sperm with the p.M138V variant, which could induce Ca^2+^ oscillations in some oocytes, as indicated in the results shown above. Previous studies have confirmed that the mRNA transcription primarily occurs at the early stage of spermatogenesis, before spermiogenesis. During their development, spermatids exchange RNA transcripts through cytoplasmic bridges, suggesting that a group of sperms may be genetically diverse yet phenotypically similar (Dadoune *et al.* 2004). As the variation p.C196* in case 5 was proved to be completely deleterious, the variation p.R391* could partially induce Ca^2+^ oscillations. The variation p.C196* may affect the function of the variation p.R391* by sharing RNA transcripts. The compound heterozygous mutations in PLCZ1 suggest a gene dosage effect on oocyte activation due to the interaction between the variation p.R391* and the variation p.C196*. The protein level of PLCZ in the sperm of the patient in case 5 increased significantly, while in HEK293T cells, the protein and transcription levels showed no obvious changes and decreased significantly following their transfection with PLCZ^M138V^ and PLCZ^C196*^. Compared with PLCZ^WT^, a novel hydrogen bond was detected between residues V138 and R584 by molecular dynamic simulation, indicating that PLCZ^M138V^ may have a more stable structure than PLCZ^WT^. In conclusion, the elevated protein level in sperm might be a result of the suppression of protein degradation caused by the inhibition of ubiquitination due to structural alterations in PLCZ (Liu *et al.* 2010). Therefore, further studies should be carried out to clarify the mechanisms behind the up-regulated protein expression in sperm with these two heterozygous mutations.

### Abnormal localisation of PLCZ in sperm of TFF patients

The sperm of patients could be divided into a few groups depending on the subcellular localisation of PLCZ, mostly in three regions: the acrosome region, post acrosomal region, and equatorial region (Dai *et al.* 2020). The abnormal ratio of subcellular localisation in the whole population of sperm cells was believed to indicate a functional alteration in PLCZ (Escoffier *et al.* 2016, Dai *et al.* 2020). Typically, the acrosome region was identified as the main subcellular location of PLCZ in the controls (the healthy individuals), while in patients with TFF due to OAF, the proportion would decrease (Escoffier *et al.* 2016, Dai *et al.* 2020). Concerns have been raised about the relationship between the structure of the acrosome (intact, reactive acrosome, and no acrosome staining) and the localisation ratio of PLCZ. The proportion of PLCZ localisation differs between sperm with intact acrosomes and entire sperm in patients (Torra-Massana *et al.* 2019). In the current study, the ratio of sperm cells from the patient in case 5 with PLCZ located at Ac decreased but increased in the unclear region, indicating a strong correlation between the localisation of PLCZ and TFF. However, future studies should be carried out to elucidate the relationship between the ability of OA and different categories of sperm based on their PLCZ location. Furthermore, the underlying mechanism of PLCZ localisation and transport to specific regions in sperm should be further investigated. Three related candidate proteins involved in PLCZ localisation have been identified by previous studies: calmodulin (CaM), actin-like protein 7A (ACTL7A), and actin-like protein 9 (ACTL9) (Thanassoulas 2022). CaM is located similarly to PLCZ at the sperm head and could form a CaM–PLCZ complex as demonstrated by the ‘pull-down’ assay and docking simulations (Leclerc *et al.* 2020). A mutation in *ACTL7A* and *ACTL9* may induce a significant reduction of *PLCZ1* expression and a lack of PLCZ in the sperm head, resulting in inadequate OA and total fertilisation failure (Xin 2020, Wang *et al.* 2021, Zhao 2023). Details about how CaM, ACTL7A, and ACTL9 coordinate PLCZ location should be further elucidated to identify additional molecular targets for the rescue of abnormal PLCZ-induced TFF.

### Functional impact of mutations in *PLCZ1* on infertility and their value in the diagnosis and treatment of infertility

Mutations in *PLCZ1* resulting in male infertility have been associated with TFF after ICSI (Torra-Massana *et al.* 2019, Cheung *et al.* 2020 Dai *et al.* 2020, Mu *et al.* 2020, Yan *et al.* 2020, Yuan *et al.* 2020, Peng *et al.* 2023, Torra-Massana *et al.* 2023). Furthermore, OAF is the main cause of TFF after ICSI (Mu *et al.* 2020). Mutations in *PLCZ1* relating to embryonic arrest and male infertility with polyspermy have also been reported (Lin *et al.* 2023, Peng *et al.* 2023). In this study, five patients suffering from TFF-linked infertility were recruited. Among them, patients in cases 1, 3, and 4 did not acquire normally fertilised zygotes in ICSI cycles, indicating OAF. The presence of normally fertilised zygotes but the absence of post-cleavage embryos in the ICSI cycle of the patient in case 2 and the presence of both normally fertilised zygotes and post-cleavage embryos in the IVF cycle of the patient in case 5 indicated the complex mechanism of *PLCZ1* mutation-related TFF, which may be related to insufficient Ca^2+^ oscillations and unexplained embryo arrest. It has been shown that *PLCZ1* functions in OA by generating Ca^2+^ transients in oocytes. The phenotype of polyspermy and early embryonic arrest and its relevance to incomplete OA need further elucidation. Furthermore, the compound mutations and the partial embryo development identified in this study indicate that in the future, advanced non-invasive sperm selection may produce more viable embryos for sperm associated with TFF, especially those carrying the heterozygous variant (Khakpour *et al.* 2019). The recently reported germline mutation profiling may provide more insights into the significance of mutation profiles in genetic diagnosis (Cheung *et al.* 2023) Understanding the relationship between *PLCZ1* mutation profiling and its function provides avenues for targeted interventions for treating TFF.

In conclusion, five variants with compound heterozygous mutations, including three mutations reported by previous studies and five novel mutations in *PLCZ1*, were identified in five patients experiencing TFF. Among the five novel mutations, one was in a region between the EF-hands domain and the X catalytic domain. Functional and conformational alterations were investigated by both *in silico* analysis and *in vitro* functional analysis, shedding light on the relevance of both function and structure of PLCZ. Additionally, a novel variant exhibiting compound heterozygosity with an elevated protein expression level was identified. This study enhances the potential for genetic diagnosis of male infertility due to TFF, thus expanding the spectrum of genetic causes associated with *PLCZ1*.

## Supplementary Materials

Supplemental Table S1. Primers used in Sanger sequencing.

Supplemental Table S2. Primers used to generated mutant plasmids with PLC ζ <sup>WT</sup> construct as template.

Supplemental Table S3. Mutations identified in PLCZ1 reported by previous studies.

Supplementary Figures 1

## Declaration of interest

The authors declare that there is no conflict of interest that could be perceived as prejudicing the impartiality of the study reported.

## Funding

This work was supported by the Natural Science Foundation of China (Grant no. 81971445).

## Ethics approval statement

The ethics was approved by the Ethical Review Board of West China Second University Hospital, Sichuan University (Approval no. 2020-031).

## Availability of data and materials statement

All relevant data will be made available upon request.

## Author contribution statement

WX, BL, and JG designed the study and modified the manuscript. QL and JG drafted the manuscript. QL, GH, and JG analysed the genetic data and performed Sanger sequencing. NW and ZJ performed the in-silico experiments. QL, GH, JG, SC, JD, XZ, JY, ZR, FY, SW, and GL performed the *in vitro* experiments and collected and analysed the data. GZ, WZ, and JZ prepared the biological samples. All authors read and approve the final version of the manuscript.
