# Supplementary material for: Novel PLCZ1 compound heterozygous mutations indicate gene dosage effect involved in total fertilisation failure after ICSI
Source: Reproduction. 2024 Sep 16;168(4):e230466. doi: 10.1530/REP-23-0466 (PMC11466203; doi:10.1530/REP-23-0466)
Supplement: Supplemental Table S1. Primers used in Sanger sequencing. [file supplementary_table_1.pdf]

Supplemental Table S1. Primers used in Sanger sequencing.

Mutation site	Forward Primer	Reverse Primer
c.941A>G (p.D314G)	GCTGAAGCGTTTACTACCAGAAG	TTTCACAAAACACCACCTCACC
c.1171C>T (p.R391*)	ATTGCTCTGGCCTTATCTGATC	TCAATCTTCTCCTGCGCACC
c.1657C>T (p.R553C)	GAATTGCCTAAGTACATCAGTC	TAATTAGCCTTCCTGTCAGTC
c.1733T>C (p.M578T)	GAATTGCCTAAGTACATCAGTC	TAATTAGCCTTCCTGTCAGTC
c.590G>A (p.R197H)	TTATATGGGTTGGGAGTAGGG	AATTGAAATGATGCATTGTTG
c.1235G>C (p.R412T)	GTGACTTCCATATGATTCTGCC	TTTACCCCGCTTCAACCCAG
c.412A>G (p.M138V)	GTGTGGTGAGTGACTGGGAAG	TGTGTTATGTGAAGATGAAAT
c.588C>A (p.C196*)	TTATATGGGTTGGGAGTAGGG	AATTGAAATGATGCATTGTTG

All data are based on NM_033123.3. (NCBI accession number) and Q86YW0 (UniProt ID).

[在此处键入]
