# Supplementary material for: Novel PLCZ1 compound heterozygous mutations indicate gene dosage effect involved in total fertilisation failure after ICSI
Source: Reproduction. 2024 Sep 16;168(4):e230466. doi: 10.1530/REP-23-0466 (PMC11466203; doi:10.1530/REP-23-0466)
Supplement: Supplemental Table S2. Primers used to generated mutant plasmids with PLC ζ WT construct as template. [file supplementary_table_2.pdf]

Supplemental Table S2. Primers used to generated mutant plasmids with PLC ζ ^{WT} construct as template.

**Mutation site**	**Forward Primer**	**Reverse Primer**
c.941A>G (p.D314G)	CATGAAAGAAAAGGTTCTGGTAAGCGTGGAGACAATC	CCAGAACCTTTTCTTTCATGGGTTTCCTTTAAGGTTC
c.1171C>T (p.R391*)	GAAAACTTTCAAAATTGTGAGTCCATGAGTTTATTTTTC	ACAATTTTGAAAGTTTTCGGGCTTGTGTCTC
c.1657C>T (p.R553C)	GAATTGGCATTGATATGTTTTGTTGTTGAAGG	ATATCAATGCCAATTCTGGGACATGAATAATAAATGTG
c.1733T>C (p.M578T)	CTTTGCCACTTCTATGCACGAACAAAGGTTATCGTC	GTGCATAGAAGTGGCAAAGTATATTGCCCAAG
c.590G>A (p.R197H)	CCCTTGTGAAAGGATGCCATTGTTTGGAG	TGGCATCCTTTCACAAGGGCACTTACATATC
c.1235G>C (p.R412T)	GAATATATCCCAAAGCAACAACAGCAGACTCTTC	GTTGTTGCTTTGGGATATATTCTGGTAATGAACTTC
c.412A>G (p.M138V)	CATTAGAAGGTTTTACAAGATACGTGGATTCACGTG	CGTATCTTGTAAAACCTTCTAATGACATTTGGTGTG
c.588C>A (p.C196*)	GCCCTTGTGAAAGGATGACGTTGTTTGG	TCATCCTTTCACAAGGGCACTTACATATCCC

[在此处键入]
