# Supplementary material for: Novel PLCZ1 compound heterozygous mutations indicate gene dosage effect involved in total fertilisation failure after ICSI
Source: Reproduction. 2024 Sep 16;168(4):e230466. doi: 10.1530/REP-23-0466 (PMC11466203; doi:10.1530/REP-23-0466)
Supplement: Supplemental Table S3. Mutations identified in PLCZ1 reported by previous studies. [file supplementary_table_3.pdf]

Supplemental Table S3. Mutations identified in *PLCZ1* reported by previous studies.

Variation Type (Homo/Het/Het$^{\Delta}$)	Mutation Site	Position In PLC Z	Genomic Location	In Silico Analysis	Function Analysis In Vitro				Pathogenicity	Phenotype of disease	Reference/S
					Catalytic activity	Function in calcium oscillations	Protein (PLC ζ)	mRNA level			
Het	c.360C>G, p.I120M	EF-hands domain	12: 18876252	√	–	Not resulted in a significant decrease (human MII oocyte)	–	–	Benign	TFF	(Torra-Massana et al., 2019)
Homo	c.588C>A, p.C196*	X catalytic domain	12: 18 865 902	√(obvious alteration of conformation)	–	Significantly lowered rates of pronucleus formation (mouse MII oocytes) (1)	Undetectable (3), abnormally localized and significantly decreased (1)	–	Pathogenic	TFF, partial fertilization failure (2), polyspermy (1)	(Dai et al., 2020, Hua et al., 2023, Peng et al., 2023, Yan et al., 2020)
Homo	c.590G>A, R197H	X catalytic domain	12: 18865900	√	–	Significantly lower rates of pronucleus formation (mouse MII oocytes)	Significantly decreased	–	Pathogenic	Partial fertilization failure	(Mu et al., 2020)
Het	c.671T>C, p.L224P	X catalytic domain	12: 18865819	√	–	Not result in a significant decrease (human MII oocyte)	–	–	Unclear	Partial fertilization failure	(Torra-Massana, et al., 2019)
Het	c.698A>T, p.H233L	X catalytic domain	12: 18865792	√	–	Partially decreased (human MII oocyte), partially decreased (mouse MII oocyte)	–	–	Pathogenic	TFF (2)	(Kashir et al., 2012, Torra-Massana, et al., 2019)
Homo	c.C736>T, p.L246F	X catalytic domain	12: 18858228	√ (affecting the hydrogen bonds)	–	–	Abnormally localized	–	Potentially pathogenic	TFF	(Dai, et al., 2020)
Het	c.972_973 delAG, p.V326K fs*25	X-Y Linker	12: 18854479_18854480	√	–	Impaired (human MII oocyte)	–	–	Pathogenic	TFF	(Torra-Massana, et al., 2019)
Homo	c.T1048C, p.S350P	Y catalytic domain	12: 18852854	√ (affecting the hydrogen bonds)	–	–	Abnormally localized	–	Potentially pathogenic	TFF	(Dai, et al., 2020)
Homo	c.1151C>T, p.A384V	Y catalytic domain	12: 18 852 751	√ (affecting the hydrogen bonds)	–	Impaired (human MII oocyte) (1)	Undetectable (2)	–	Pathogenic	TFF (1), polyspermy (1)	(Lin et al., 2023, Yan, et al., 2020)
Het	c.1193A>C, p.H398P	Y catalytic domain	12: 18849182	√ (affecting the hydrogen bonds)	–	Partially decreased and number of spikes decreased (mouse MII oocytes) (1), impaired (mouse MII oocytes)	–	–	Pathogenic	–	(Heytens et al., 2009, Nomikos et al., 2011)
Homo	c.1465A>T, p.I489F	C2 domain	12: 18841149	√ (affecting the C2 domaina and C2/EF-hand junction)	No impact, affecting the binding of PLC ζ to PI(3)P- and PI(5) P-containing liposomes (1)	Partially decreased and number of spikes decreased (mouse MII oocytes) (2)	Undetectable (1), no impact (1)	–	Pathogenic	TFF (2)	(Escoffier et al., 2016)
Homo, Het	c.1499C>T, p.S500L	C2 domain	12: 18841115	√	–	Not resulted in a significant decrease (human MII oocyte)	–	–	Unclear	Partial fertilization failure (Homo (1 patient), Het (4 patients, one of them were not OAF); TFF (Het 3 patients);	(Torra-Massana, et al., 2019)
Homo	c.C1607>T, p.W536X	C2 domain	12: 18837198	√	–	–	Undetectable	–	Unclear	Early embryos arrested	(Lin, et al., 2023)
Homo	c.1658 G>C, p. R553P	C2 domain	12: 18837147	√ (affecting the hydrogen bonds)	–	Significantly lowered rates of pronucleus formation (human MII oocyte)	No impact	–	Pathogenic	TFF	(Yuan et al., 2020)
Homo	c.1727T>C, p.L576P	C2 domain	12: 18 837 078	√(affecting C2/catalytic domain interaction)	Significantly impaired	–	–	No impact	Unclear	Partial fertilization failure	(Yuan et al., 2020)

Het$^{\Delta}$	c.1174+3A>C, –	–	12: 18852725	√ (splice site change and affected protein features)	–	–	–	Significantly decreased	Pathogenic	TFF	(Zhao S, 2023)
	c.A1274>G, p.N425S	Y catalytic domain	12: 18849101	√ (affecting the hydrogen bonds)	–	–	–	Significantly decreased	Pathogenic		(Zhao S, 2023)
Het$^{\Delta}$	c.136-1G>C, –	–	12: 18876476	√ (splice site change and affected protein features)	–	–	–	–		TFF	(Zhao S, 2023)
	c.G1358A, p.G453D	Y catalytic domain	12: 18847948	√ (affecting the hydrogen bonds)	–	–	–	–	Unclear		(Zhao S, 2023)
Het$^{\Delta}$	c.830T>C, p.L277P	X catalytic domain	12: 18 858 134	√(affecting the catalytic domain)	–	Decreased proportion (human MII oocyte)	Undetectable	–	Pathogenic	TFF	(Yan, et al., 2020)
	c.588C>A, p.C196*	X catalytic domain	12: 18 865 902	√	–	–	Undetectable	–	Pathogenic		(Yan, et al., 2020)
Het$^{\Delta}$	c.1129_1131delAAT, p.N377del	Y catalytic domain	12: 18852771_18852773	√ (affecting the core region of the catalytic domain)	–	Impaired (human MII oocyte)	Undetectable	–	Pathogenic	TFF	(Yan, et al., 2020)
	c.1733T>C, p.M578T	C2 domain	12: 18 837 072	√ (severely destabilize the hydrophobic core)	–	Impaired (human MII oocyte)	Undetectable	–	Pathogenic		(Yan, et al., 2020)
Het$^{\Delta}$	c.570+1G>T, p.V189Cfs*12	X catalytic domain	12: 18 872 364	√	–	–	Undetectable	–	Pathogenic	Partial fertilization failure	(Yan, et al., 2020)
	c.1344A>T, p.K448N	Y catalytic domain	12: 18 847 961	√ (cause a potential instability of the interaction between the catalytic domain and the C2 domain.)	–	Partially decreased (human MII oocyte)	Undetectable	–	Pathogenic		(Yan, et al., 2020)
Het$^{\Delta}$	c.1259C>T, p.P420L	Y catalytic domain	12: 18849116	√(affecting helix and structural stability)	Significantly impaired	–	–	No impact	Pathogenic	Tff	(Yuan, et al., 2020)
	c.1733T>C, p.M578T	C2 domain	12: 18 837 072	√ (severely destabilize the hydrophobic core)	Significantly impaired	–	–	No impact	Pathogenic		(Yuan, et al., 2020)
Het$^{\Delta}$	c.590G>A, p.R197H	X catalytic domain	12: 18865900	√	–	A decrease in the percentage of activated oocytes (human MII oocyte)	–	–	Pathogenic	TFF	(Torra-Massana, et al., 2019)
	c.1499C>T, p.S500 L	C2 domain	12: 18841115	√	–	Not resulted in a significant decrease (human MII oocyte)	–	–	Unclear		(Torra-Massana, et al., 2019)
Het$^{\Delta}$	c.698A>T, p.H233L	X catalytic domain	12: 18865792	√(obvious alteration of conformation)	–	Decreased proportion (human MII oocyte)	–	–	Pathogenic	TFF	(Kashir, et al., 2012)
	c.1193A>C, p.H398P	Y catalytic domain	12: 18849182	√	–	–	–	–	Pathogenic		(Kashir, et al., 2012)
Het$^{\Delta}$	c.590G>A, R197H	X catalytic domain	12: 18865900	√ (affecting the hydrogen bonds)	–	–	Abnormally localized and significantly decreased	–	Pathogenic	TFF	(Peng, et al., 2023)
	c.2 T > C, p.M1T	–	12: 18890304	√(affecting helix or the hydrogen bonds)	–	–	Abnormally localized and significantly decreased	–			(Peng, et al., 2023)
Het$^{\Delta}$	c.588C>A, p.Cys196*	X catalytic	12: 18865902	√	–	Significantly lowered rates of pronucleus	Undetectable	–	Pathogenic	Partial fertilization failure	(Mu, et al., 2020)

		domain				formation (mouse MII oocytes)					
	c.1259C>T, p.P420L	Y catalytic domain	12: 18849116	√	–	Significantly lower rates of pronucleus formation (mouse MII oocytes)	Significantly decreased	–	Pathogenic		(Mu, et al., 2020)
Het$^{\Delta}$	c.972_973delAG , p.T324fs	X-Y Linker	12: 18854480	–	–	Significantly lower rates of pronucleus formation (mouse MII oocytes)	Truncated	–	Pathogenic	TFF	(Mu, et al., 2020)
	c.1234delA, p.R412fs	Y catalytic domain	12: 18849141	–	–	Significantly lower rates of pronucleus formation (mouse MII oocytes)	Truncated	–	Pathogenic		(Mu, et al., 2020)
Het$^{\Delta}$	c.588C>A, p.Cys196*	X catalytic domain	12: 18865902	√	–	–	Undetectable	–	Pathogenic	Polyspermy	(Lin, et al., 2023)
	c.T1466>G, p.I489S	C2 domain	12: 18841149	√	–	–	Undetectable	–	Pathogenic		(Lin, et al., 2023)
Het$^{\Delta}$	c.588C>A, p.Cys196*	X catalytic domain	12: 18865902	√	–	–	Undetectable	–	Pathogenic	Early embryos arrested	(Lin, et al., 2023)
	c.1208_1213del, p.403_404del	Y catalytic domain	12: 18849171	√	–	–	undetectable	–	–		(Lin, et al., 2023)

Homo: Homozygosity, Het: Heterozygosity, Het$^{\Delta}$: Compound heterozygosity; The total numbers of patients in which the specific variant had been identified or with whom the phenotype of disease had been diagnosed and the total number of mutations which exhibited specific activity during in silico analysis and in vitro function analysis were shown within parentheses.

References

Dai J, Dai C, Guo J, Zheng W, Zhang T, Li Y, Lu C, Gong F, Lu G, Lin G. Novel homozygous variations in PLCZ1 lead to poor or failed fertilization characterized by abnormal localization patterns of PLCzeta in sperm. *Clin Genet* 2020;97: 347-351.

Escoffier J, Lee HC, Yassine S, Zouari R, Martinez G, Karaouzene T, Coutton C, Kherraf ZE, Halouani L, Triki C *et al.* Homozygous mutation of PLCZ1 leads to defective human oocyte activation and infertility that is not rescued by the WW-binding protein PAWP. *Hum Mol Genet* 2016;25: 878-891.

Heytens E, Parrington J, Coward K, Young C, Lambrecht S, Yoon SY, Fissore RA, Hamer R, Deane CM, Ruas M *et al.* Reduced amounts and abnormal forms of phospholipase C zeta (PLCzeta) in spermatozoa from infertile men. *Hum Reprod* 2009;24: 2417-2428.

Hua R, Xue R, Liu Y, Li Y, Sha X, Li K, Gao Y, Shen Q, Lv M, Xu Y *et al.* ACROSIN deficiency causes total fertilization failure in humans by preventing the sperm from penetrating the zona pellucida. *Human Reproduction* 2023;38: 1213-1223.

Kashir J, Konstantinidis M, Jones C, Lemmon B, Lee HC, Hamer R, Heindryckx B, Deane CM, De Sutter P, Fissore RA *et al.* A maternally inherited autosomal point mutation in human phospholipase C zeta (PLCzeta) leads to male infertility. *Hum Reprod* 2012;27: 222-231.

Lin Y, Huang Y, Li B, Zhang T, Niu Y, Hu S, Ding Y, Yao G, Wei Z, Yao N *et al.* Novel mutations in PLCZ1 lead to early embryonic arrest as a male factor. *Front Cell Dev Biol* 2023;11: 1193248.

Mu J, Zhang Z, Wu L, Fu J, Chen B, Yan Z, Li B, Zhou Z, Wang W, Zhao L *et al.* The identification of novel mutations in PLCZ1 responsible for human fertilization failure and a therapeutic intervention by artificial oocyte activation. *Mol Hum Reprod* 2020;26: 80-87.

Nomikos M, Elgmati K, Theodoridou M, Calver BL, Cumbes B, Nounesis G, Swann K, Lai FA. Male infertility-linked point mutation disrupts the Ca2+ oscillation-inducing and PIP(2) hydrolysis activity of sperm PLCzeta. *Biochem J* 2011;434: 211-217.

Peng Y, Lin Y, Deng K, Shen J, Cui Y, Liu J, Yang X, Diao F. Mutations in PLCZ1 induce male infertility associated with polyspermy and fertilization failure. *J Assist Reprod Genet* 2023;40: 53-64.

Torra-Massana M, Cornet-Bartolome D, Barragan M, Durban M, Ferrer-Vaquer A, Zambelli F, Rodriguez A, Oliva R, Vassena R. Novel phospholipase C zeta 1 mutations associated with fertilization failures after ICSI. *Hum Reprod* 2019;34: 1494-1504.

Yan Z, Fan Y, Wang F, Yan Z, Li M, Ouyang J, Wu L, Yin M, Zhao J, Kuang Y *et al.* Novel mutations in PLCZ1 cause male infertility due to fertilization failure or poor fertilization. *Hum Reprod* 2020;35: 472-481.

Yuan P, Yang C, Ren Y, Yan J, Nie Y, Yan L, Qiao J. A novel homozygous mutation of phospholipase C zeta leading to defective human oocyte activation and fertilization failure. *Hum Reprod* 2020;35: 977-985.

Yuan P, Zheng L, Liang H, Lin Q, Ou S, Zhu Y, Lai L, Zhang Q, He Z, Wang W.

Novel mutations in the PLCZ1 gene associated with human low or failed fertilization. *Mol Genet Genomic Med* 2020;8: e1470.

Zhao S CY, Guo S, Liu B, Bian Y, Zhao S, Chen Z, Zhao H. Novel variants in ACTL7A and PLCZ1 are associated with male infertility and total fertilization failure. *Clin Genet* 2023;103: 603-608.
