# Supplementary material for: Novel PLCZ1 compound heterozygous mutations indicate gene dosage effect involved in total fertilisation failure after ICSI
Source: Reproduction. 2024 Sep 16;168(4):e230466. doi: 10.1530/REP-23-0466 (PMC11466203; doi:10.1530/REP-23-0466)
Supplement: Supplementary Figures 1 [file supplementary_figure_1.pdf]

A

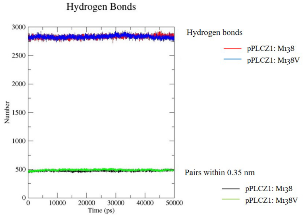

B

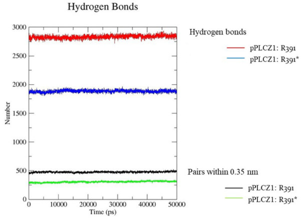

C

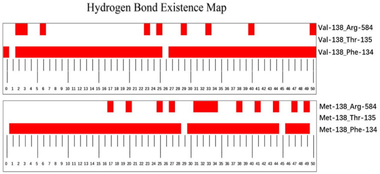

Hydrogen bond	Met-138_Phe-134	Met-138_Thr-135	Met-138_Arg-584
Occupancy	93%	2%	27%
Hydrogen bond	Val-138_Phe-134	Val-138_Thr-135	Val-138_Arg-584
Occupancy	94%	1%	26%

D

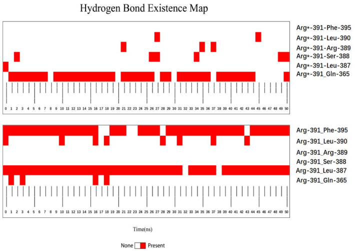

Hydrogen bond	Arg-391_Gln-365	Arg-391_Leu-387	Arg-391_Ser-388	Arg-391_Arg-389	Arg-391_Leu-390	Arg-391_Phe-395
Occupancy	9%	92%	0%	0%	8%	94%
Hydrogen bond	Arg*-391_Gln-365	Arg*-391-Leu-387	Arg*-391-Ser-388	Arg*-391-Arg-389	Arg*-391-Leu-390	Arg*-391-Phe-395
Occupancy	72%	0%	8%	8%	4%	0%
